# Need to Understand the Gaur Ecology and Methodological Framework: A Reply to Sharma (2025)

**DOI:** 10.1002/ece3.73575

**Published:** 2026-04-29

**Authors:** Bishnu Prasad Bhattarai, Hem Bahadur Katuwal, Sandeep Regmi, Bishnu Aryal, Krishna Tamang, Sabin KC, Amrit Nepali, Shivish Bhandari, Amir Basnet, Pradip Kandel, Chandu Paneru, Bishal Subedi, Niraj Regmi, Sabina Koirala, Haribhadra Acharya, Jerrold L. Belant, Hari Prasad Sharma

**Affiliations:** ^1^ Central Department of Zoology Institute of Science and Technology, Tribhuvan University Kirtipur, Kathmandu Nepal; ^2^ Nepal Zoological Society Kirtipur, Kathmandu Nepal; ^3^ Center for Integrative Conservation, Xishuangbanna Tropical Botanical Garden, Chinese Academy of Sciences Mengla Yunnan China; ^4^ Natural Science Society Kirtipur, Kathmandu Nepal; ^5^ The Himalayan Conservancy Kirtipur, Kathmandu Nepal; ^6^ Key Laboratory of Ecological Safety and Sustainable Development in Arid Lands Xinjiang Institute of Ecology and Geography, Chinese Academy of Sciences Urumqi China; ^7^ Department of National Parks and Wildlife Conservation Babarmahal, Kathmandu Nepal; ^8^ Department of Fisheries and Wildlife Michigan State University East Lansing Michigan USA

Recently, Sharma (2025) commented to our article Bhattarai et al. (2025) regarding our results reflecting gaur ecology or could be influenced in part by our methodological approach. We appreciate the opportunity to provide clarification on the apparent misunderstandings raised in this letter. We suggest the concerns raised by the authors reflect a misunderstanding of the research design, sampling approach, and ecological context of gaur and Parsa National Park. Nonetheless, we welcome the opportunity to clarify these aspects and trust that our response will help address the concerns raised.

1. Sharma (2025) commented on our hypothesis that gaur occupancy decreases in areas with higher tiger density due to predation risk, citing Hayward et al. (2012) to suggest that tigers avoid gaur. Although tigers are known to prefer medium‐sized prey (Hayward et al. 2012), multiple studies document predation on gaur by tigers. For example, Andheria et al. (2007) reported that gaur occurred in 27.82% of tiger scats and contributed 42.31% of the relative biomass. In addition, the gaur constitutes a major component of the tiger's diet in areas where both species coexist (Bhandari et al. 2025). Similarly, Variar et al. (2023) found a 14% frequency of occurrence with a 14.48% contribution to relative biomass. Although these values are lower than that of medium‐sized prey, they show that tigers do consume large prey such as gaur. Moreover, Krishnakumar et al. (2022) observed strong dietary selectivity for gaur (Jacob's *D* = 0.937), with gaur comprising 75% of total biomass consumed. It is also worth noting that Hayward et al. (2012) did not state that tigers significantly avoid gaur. The species identified as significantly avoided were peafowl (*Z* = 100, *n* = 6, *p* = 0.001), langur (*t*_(26)_ = −15.06, *p* < 0.001), and nilgai (*t*_(32)_ = −2.35, *p* = 0.025). Including tiger presence in ecological analyses can reveal spatial avoidance and fine‐scale habitat use by gaur, which is important for understanding landscape‐level predator and prey dynamics. We therefore recommend drawing inferences from multiple sources rather than relying on a single study.
2. Sharma (2025) also questioned our methodology for testing the hypothesis that gaur occupancy declines with increasing distance from water, suggesting that cameras should be placed at varying distances from water sources. This recommendation overlooks the distribution of waterbodies and the constraints of systematic sampling in our study area. The park contains numerous waterbodies, and it is not feasible to position each camera with reference to a specific water source. Instead, we deployed 67 cameras on a 20 5 × 5 km grid, which naturally yielded a wide gradient of distances to water across the landscape. As reported in the manuscript, “the mean distance to the nearest permanent water source was 3526.2 ± 2578.5 m.” If distances had been uniform, reporting a mean and standard deviation would have had little value; the observed variability reflects real field conditions. Because our survey was conducted in winter, some waterbodies may have likely dried, further contributing to spatial and temporal variation in water availability. More broadly, in natural systems, it is rarely possible (or desirable) to locate all sampling sites at equal distances from key features. Crucially, distance‐based inference benefits from sampling across the gradient of conditions within the study area or system of inference when spatial covariates are modeled as continuous predictors, which is how we analyzed distance to water in our occupancy models.
3. Sharma (2025) questioned our explanation for why the number of camera traps per grid cell ranged from one to four. Our intent was to place four cameras in each cell, but this was not always feasible because of field conditions and access constraints, as we further describe below. To summarize spatial coverage, we used the convex hull polygon of all camera‐trap locations to delineate the surveyed extent. Geographic coordinates for each station are provided on the map, and distances were derived from these GPS waypoints in a GIS workflow. Once a camera is deployed at a known latitude and longitude, distances to features of interest can be calculated accurately. As stated in the Methods, cameras were placed along walking trails with a minimum spacing of 1 km, and it will be obvious that GPS will be used for it.

The author further stated that many camera locations appear linear, with some areas overrepresented and others underrepresented, and that we avoided the northern side of the park despite reports that gaur use hilly terrain up to 2800 m elevation (Ashokkumar et al. 2011). Our design prioritized safe and repeatable access along established trails, which is common practice in dense forests and enhances both detectability and equipment security. We did not exclude the northern sector and several cameras were deployed there as logistics allowed. The comparison to Ashokkumar et al. (2011) is not directly applicable. Parsa National Park reaches approximately 950 m above sea level, and our camera placements extended to about 560 m, reflecting the park's higher elevation that we could access. Conducting systematic sampling off trail in steep, closed‐canopy forest is both logistically challenging and potentially unsafe.

Finally, according to the Gaur Conservation Action Plan of Nepal, the population in Parsa National Park is estimated at roughly 105 individuals, concentrated primarily in lower grasslands. Our allocation of effort therefore emphasized areas where gaur most likely occurred, while still spanning the park's environmental and spatial gradients. In summary, the variation in cameras per grid reflected real‐world constraints and the spatial arrangement and distances were documented and handled explicitly in our analyses.

4 Sharma (2025) suggests that because we analyzed each camera station independently, the grid was unnecessary. We used the 5 × 5 km grid as a design‐based spatial framework to prevent clustering, distribute effort across habitats, and better ensure uniform coverage. Within that framework, each camera station served as a site with its own detection history, which allowed the analysis to capture local heterogeneity in habitat, water availability, and predator presence. These two elements are complementary rather than redundant: the grid controls spatial placement and effort, whereas the point‐level (i.e., camera‐level) analysis estimates occupancy at the scale at which animals interact with the landscape.
5 Sharma (2025) notes that detection heterogeneity is often modeled in occupancy studies (Karavarsamis and Huggins 2019), we did not include site‐ or habitat‐level covariates for detection, and that we used inverse distance weighting (IDW) to map detection probability, which can be sensitive to clustered samples (Benmoshe 2025). We agree that modeling detection with covariates is common practice when suitable predictors are available. In our case, we specified a constant detection probability because the survey window was short and the population was assumed closed during sampling. This is a standard and defensible assumption in occupancy analyses. As stated in the Methods, we interpreted occupancy as an indicator of habitat use rather than “true” occupancy (Sunarto et al. 2012; Gould et al. 2019). In addition, the Bayesian hierarchical framework provides partial pooling and can accommodate latent variation even when explicit detection covariates are limited (Royle and Dorazio 2008).

Regarding the IDW map, our intent was for a descriptive visualization of spatial patterns in detections, not inference. Although IDW can be sensitive to clustered inputs, our grid‐based deployment and minimum spacing reduced clustering, and all inference was drawn from the occupancy model rather than the interpolated surface. We used IDW to help readers visualize spatial autocorrelation evident in the data; use of IDW does not affect parameter estimation or the conclusions of the model.

6 The commenter suggested a lack of clarity about the elephant detection variable. However, we clearly state in the abstract that elephant presence was included as a covariate in the gaur occupancy model, which we also described in the Methods, reported on in the Results, and interpreted in the Discussion. For brevity, elephant presence was derived from camera detections during the same sampling period and used as a site‐level covariate in the occupancy analysis.
7 Sharma (2025) states that there was no model checking, but we did assess model accuracy. Within the Bayesian framework used, we examined posterior credible intervals to evaluate the plausibility of parameter estimates and predictions given the data. We also confirmed MCMC convergence using the Gelman–Rubin diagnostic, applying a threshold of *R̂* < 1.1 (Gelman and Rubin 1992; Brooks and Gelman 1998). Full details are provided in the Data Analysis section of Bhattarai et al. (2025).
8 Sharma (2025) argues that credible intervals are obscured and that the wide intervals shown in the figure make it risky to generalize to the entire gaur population of Parsa National Park. As commenter said, we report the credible intervals in the results and explicitly cautioned against overgeneralization in the Discussion: “However, these results should be interpreted with caution, as finer‐scale habitat assessments and long‐term monitoring could reveal more nuanced ecological interactions influencing gaur distribution.” Wide credible intervals reflect uncertainty arising from sample size and spatial heterogeneity; they do not imply that the results are invalid.
9 Issues with interpretation:
  - Sharma (2025) mentioned a typographical error in our statement about the positive association between Gaur habitat use and distance from water. The sentence is correct as written. A positive association means greater use farther from permanent water, which could occur to reduce competition and predation risk near water sources.
  - The author further stated that we ignore the credible intervals for interpreting the responses to forest area and elephant detection. Our study is not methodological; it focuses on biological interpretation as supported by statistical evidence described in Bhattarai et al. (2025).
  - The author mentioned that since sampling was restricted to the plains area, they lacked sufficient evidence to make the claim that gaur has restricted distribution in the Chure. Our conclusion for the Chure is based on camera detections from both accessible passes between Bhata and Pratappur during the study period. The Chure in Parsa National Park has limited access and difficult topography, so we did not cover the entire area. One co‐author is the Chief Warden of the park and provided detailed knowledge of gaur distribution that aligns with our detections. We emphasize that our inference is limited to the surveyed portion of the Chure during the period monitored only.
  - The author mentioned that our sampling strategy was inappropriate to make claims about the response to distance from water bodies. Please see our earlier response on camera deployment. We modeled distance to water as a continuous covariate across the park. The design did not require placing cameras at waterholes. Multiple factors shape species occurrence in nature, which is why we recorded several covariates that we considered potentially influential based on scientific literature and analyzed their joint effects. We are examining specific effects of these factors on gaur activity in future work.
  - The author mentioned that there is no evidence of better use of forest edges or grasslands and its relation to distance to waterbodies. We agree that gaur needs water, especially in the dry season. Distance to permanent water in our models is a spatial predictor of habitat use, not a claim that gaur avoid water. Some waterbodies likely dry seasonally, which can influence gaur space use as related to distance to water. The effects of factors influencing gaur occupancy are unlikely to be spatially uniform, so identifying spatial variation is important for management.

Overall, we appreciate Sharma (2025) for engaging with our work. Several of his points reflect misunderstandings of gaur ecology, challenges of field work, and the analytical framework used, which we have clarified above. Our main conclusions about gaur habitat use remain supported by the data and analyses presented. We agree that longer‐term, multi‐season monitoring, and finer‐scale assessments would further refine these inferences.

## Author Contributions

**Bishnu Prasad Bhattarai:** writing – review and editing (equal). **Hem Bahadur Katuwal:** writing – review and editing (equal). **Sandeep Regmi:** writing – review and editing (equal). **Bishnu Aryal:** writing – review and editing (equal). **Krishna Tamang:** writing – review and editing (equal). **Sabin KC:** writing – review and editing (equal). **Amrit Nepali:** writing – review and editing (equal). **Shivish Bhandari:** writing – review and editing (equal). **Amir Basnet:** writing – review and editing (equal). **Pradip Kandel:** writing – review and editing (equal). **Chandu Paneru:** writing – review and editing (equal). **Bishal Subedi:** writing – review and editing (equal). **Niraj Regmi:** writing – review and editing (equal). **Sabina Koirala:** writing – review and editing (equal). **Haribhadra Acharya:** writing – review and editing (equal). **Jerrold L. Belant:** writing – review and editing (equal). **Hari Prasad Sharma:** conceptualization (equal), validation (equal), writing – original draft (equal), writing – review and editing (equal).

## Conflicts of Interest

The authors declare no conflicts of interest.

## Data Availability

No primary data were collected for this manuscript.
